# Dialysis Provision and Implications of Health Economics on Peritoneal Dialysis Utilization: A Review from a Malaysian Perspective

**DOI:** 10.1155/2017/5819629

**Published:** 2017-10-31

**Authors:** Mohd Rizal Abdul Manaf, Naren Kumar Surendra, Abdul Halim Abdul Gafor, Lai Seong Hooi, Sunita Bavanandan

**Affiliations:** ^1^Department of Community Health, Faculty of Medicine, Pusat Perubatan Universiti Kebangsaan Malaysia, Jalan Yaacob Latif, Bandar Tun Razak, 56000 Cheras, Kuala Lumpur, Malaysia; ^2^Nephrology Unit, Faculty of Medicine, Pusat Perubatan Universiti Kebangsaan Malaysia, Jalan Yaacob Latif, Bandar Tun Razak, 56000 Cheras, Kuala Lumpur, Malaysia; ^3^Hospital Sultanah Aminah, Jalan Persiaran Abu Bakar Sultan, 80100 Johor Bahru, Johor, Malaysia; ^4^Hospital Kuala Lumpur, Jalan Pahang, 50586 Kuala Lumpur, Malaysia

## Abstract

End-stage renal disease (ESRD) is managed by either lifesaving hemodialysis (HD) and peritoneal dialysis (PD) or a kidney transplant. In Malaysia, the prevalence of dialysis-treated ESRD patients has shown an exponential growth from 504 per million population (pmp) in 2005 to 1155 pmp in 2014. There were 1046 pmp patients on HD and 109 pmp patients on PD in 2014. Kidney transplants are limited due to lack of donors. Malaysia adopts public-private financing model for dialysis. Majority of HD patients were treated in the private sector but almost all PD patients were treated in government facilities. Inequality in access to dialysis is visible within geographical regions where majority of HD centres are scattered around developed areas. The expenditure on dialysis has been escalating in recent years but economic evaluations of dialysis modalities are scarce. Evidence shows that health policies and reimbursement strategies influence dialysis provision. Increased uptake of PD can produce significant economic benefits and improve patients' access to dialysis. As a result, some countries implemented a PD-First or Favored Policy to expand PD use. Thus, a current comparative costs analysis of dialysis is strongly recommended to assist decision-makers to establish a more equitable and economically sustainable dialysis provision in the future.

## 1. Introduction

Malaysia is a federation of 13 states and 2 territories in a parliamentary democracy, with the Prime Minister the head of government and a constitutional monarch elected by the Conference of Rulers. Malaysians make up 0.4% of the world's total population at 31 million with gross domestic product (GDP) at US$272 billion in 2015 [1]. Life expectancy for newborn baby boy and girl was 72.6 years and 77.2 years, respectively [2]. Malaysia has a dual-tiered system of healthcare services consisting of a government-led public sector and a coexisting private sector creating a dichotomous yet synergistic public-private model [3]. The total health expenditure in 2013 was 4.53% of GDP (US$ 14, 205.7 million) [4].

Both developing and developed nations may have an ageing population with modifiable lifestyle risk factors causing chronic diseases particularly chronic kidney disease (CKD) [5]. CKD is characterized by progressive, irreversible kidney function deterioration culminating in end-stage renal disease (ESRD), which requires treatment by renal replacement therapy (RRT), either hemodialysis (HD) or peritoneal dialysis (PD) when kidney transplantation is limited or contraindicated. HD is usually performed at hospital or separate dialysis unit three times per week or sometimes at home. PD is administered at home and several PD modalities are available. The most common is continuous ambulatory PD (CAPD). Total ESRD patients were 3,200,000 but only 2,519,000 were being treated in 2013 with approximately 7% annual growth rate [6].

CKD is a global health threat with socioeconomic and public health consequences. Estimates on the Global Burden of Disease (GBD) indicated that kidney diseases were responsible for 2,993,000 years of life lost (YLL) and 38,104,000 disability adjusted life years (DALYs) lost globally [7]. In Malaysia, kidney disease ranked 8th from ten causes of death with 365.7 (YLL) per 100,000 population which accounted for 2.3% of total premature deaths [8]. Persons with CKD and ESRD have poor health related quality of life as compared to the general population [9–11]. Kidney transplantation is the best RRT option. However, the new kidney transplants' rate in Malaysia is very low at 3 per million population (pmp) due to organ shortage [12].

In addition, the healthcare costs and economic burden of CKD are huge [13]. The expenditure for the management of patients with ESRD in developed countries accounted for 2-3% of total healthcare expenditure, while ESRD patients represent only 0.02–0.03% of the total population [14]. PD is known as the most cost-effective dialysis modality in most developed countries and some developing countries [15, 16]. However, PD is underutilized around the world [17]. This article aims to review the dialysis provision, issues, and implications of health economics on PD utilization from a Malaysian perspective.

## 2. Methodology

A review of dialysis provision, issues, and implications of health economics on PD utilization was conducted from a Malaysian perspective. The 22nd Malaysian Dialysis and Transplant Registry report and other published articles through limited literature search on key resources including PubMed, Medline, and a focused Internet search were used in this review.

## 3. Results

### 3.1. Dialysis Provision in Malaysia

Malaysian Dialysis and Transplant Registry (MDTR) collects information on patients with ESRD on RRT in Malaysia. Hence, most of the data on dialysis provision are from the registry's annual report. The latest report published is the 22nd MDTR 2014 report [12]. The acceptance rate of dialysis patients had increased since 2004 with 203 pmp new HD cases and 31 pmp new PD cases in 2014. A total of 31,497 HD patients and 3,270 PD patients were dialyzing in 2014 giving a prevalence rate of 1046 pmp and 109 pmp, respectively. The dialysis treatment rate exceeded 100 pmp for all states in Malaysia except Sabah with the lowest rates in Kelantan and Sabah. In terms of gender, the treatment gap between men and women accepted for dialysis had remained constant over the years with male 55% and female 45%. Meanwhile, 58% of new dialysis patients were 55 years or older at the onset of dialysis and 61% of new patients had diabetes mellitus as the primary renal disease [12].

An influx of dialysis patients had resulted in an increase of dialysis centres especially in the private sector [18]. The number of dialysis centres for the whole of Malaysia increased from 205 in 2000 to 758 in 2014, mainly contributed by private dialysis centres which had almost tripled from 6 pmp in 2005 to 14 pmp in 2014. Nongovernmental organization (NGO) centres had only increased from 4 pmp in 2005 to 5 pmp in 2014. Meanwhile, the rate was stagnant in the public sector, 5 pmp in 2005 and 2014 [12]. Private dialysis centres are mainly distributed in economically developed west coast states of Peninsular Malaysia. The government operates most of the dialysis centres in less developed states. Majority of HD patients were in the private sector (54%) but almost all PD patients were treated in government facilities (97%) [12].

### 3.2. Survival of Dialysis Patients in Malaysia

There were 4,015 dialysis deaths reported in 2014. Modality specific death rate over the last 10 years ranged from 12 to 13% for HD and 16 to 18% for PD. The annual death rate among HD patients remained relatively unchanged while the annual death rate of PD patients began to increase in mid-2000s and appeared to have improved over the last 3 years [12].

The apparent survival difference between PD and HD patients in Malaysia began to widen after the first year. The overall unadjusted 5-year and 10-year patient survival for all dialysis was 54% and 30%, respectively. At 10 years the unadjusted patient survival on HD was 31% compared with 27% for those on PD. There were various factors associated with better patient survival including younger age and absence of diabetes. It is important to highlight that the persistent difference in annual death rate between the two modalities was partly associated with negative selection of patients for PD. After adjustments patients on PD have a 4.8% lower mortality risk compared to those on HD [12].

### 3.3. Dialysis Funding in Malaysia

Malaysia has a mixed healthcare financing system. Public healthcare services are funded through general taxation, with annual health budgets allocated by Ministry of Finance (MOF) to the MOH. Within the private sector, individuals can purchase health insurance on voluntary basis, with variable premiums charged based on their health status and the level of coverage or covered by negotiated packages with Managed Care Organizations (MCOs) and private insurance companies [19]. Civil servants and their dependents would be reimbursed by the government. Social Security Organization (SOCSO), a government-run social insurance body that receives mandatory contributions from private-sector employees earning below US$950 per month and the state-run Islamic social welfare organizations reimburse eligible patients for certain treatments and dialysis, was included as a rehabilitation therapy [18].

The government is the main source of funding for dialysis therapy for new and existing patients from 2004 to 2014. Out of pocket payment or self-funding for dialysis was about 26 to 30% and funding from NGOs remained at 11–15% over the years [12].

### 3.4. Issues Related to Dialysis Provision in Malaysia

The increasing prevalence of ESRD patients in Malaysia is of concern. Some options that have been proposed to tackle this issue include early medical intervention to slow the progression of CKD in high-risk patients, promotion of kidney transplantation, and use of the most cost-effective dialysis therapy that can be offered to a patient without compromising outcome [10, 20–22]. In Malaysia, renal failure prevention initiatives are carried out nationwide including patient screening at primary care settings, prevention of renal failure workshops targeting primary care doctors and allied healthcare staff, development of clinical practice guidelines (CPG) on CKD management and nephrology services operational policy [23], and national public awareness for World Kidney Day. Despite all these efforts, the ESRD population is increasing at an alarming rate. As kidney transplants are limited due to lack of donors, the choice of RRT lies between HD and PD.

Analysis of the expenditure of dialysis provision in Malaysia has been limited to a few studies. Total spending on dialysis in 2005 was US$ 100 million accounted for 1.72% of the total health expenditure [18]. A recent forecast data shows that the estimated cost incurred to treat 51,269 patients with dialysis in the year 2020 is US$ 384.5 million [24]. This burden will have implications for healthcare financing in the future.

Although PD is associated with lower costs than HD, it is underutilized around the world including Malaysia. Only 9% of ESRD patients are on PD and 97% of them are treated in MOH [12]. An economic evaluation study of centre HD and CAPD among MOH hospitals in Malaysia published in 2005 found that CAPD was marginally more cost-effective than HD (US$ 8325 versus US$ 8853 life year saved) [25]. PD also offer a number of medical advantages over HD including better preservation of residual renal function and less requirement for Erythropoiesis Stimulating Agents (ESAs) [26, 27]. Many nephrologists believe that home dialysis including PD is better for the patients as opposed to their current practice [28, 29].

Although dialysis treatment rates reached a level comparable to rates in developed countries [18], there is an inequitable distribution. Economically developed west coast states of Peninsular Malaysia have higher rates compared to east coast states, Sabah and Sarawak. Federal territory of Kuala Lumpur has the hugest dialysis rate at 1741 pmp and Federal Territory of Labuan and state of Sabah has the lowest dialysis rate at 486 pmp [12]. Registries capture information on patients who are on RRT, not all those who develop ESRD. Recently published article show that there is a gap between incidence of ESRD and use of RRT which was estimated through the prevalence of diabetes and hypertension [30, 31]. In Malaysia, overall prevalence of hypertension and diabetes (known and undiagnosed) among adults of 18 years and above was 30.3% and 17.5%, respectively [32]. In a population-based study by Hooi et al., it was reported that the prevalence of CKD stage 5 in Malaysia was 0.36% in people above 18 years old [33]. However, only 0.17% of people above 18 years old were on RRT at the same year; that is, about half of the CKD stage 5 were on RRT. Although some patients might not be suitable for dialysis or do not need it yet (dialysis is started at about GFR 5–7 ml/min, CKD stage 5 is GFR < 15 ml/min) or die before dialysis is started, a gap is observed between CKD stage 5 and RRT provision in Malaysia.

### 3.5. Factors Influencing Selection of Dialysis Modalities

Medical and nonmedical factors including social conditions, geographic considerations, economic/reimbursement, and patient choice dictate the selection of PD or HD, with patient choice being an important factor [34]. In Malaysia, the selection of patients for long-term hemodialysis in MOH is under the purview of a selection committee according to selection criteria including waiting time and priority group (failed graft following kidney transplant at MOH hospitals, children, change of modality) [23]. The criteria for selection of patients for long-term PD include patient choice with absence of contraindications to PD. There are factors to consider including vision, manual dexterity and availability of assistant, home environment, and lack of vascular access [23].

Although many factors affect the selection of one dialysis modality, absolute medical contraindications for the use of either modality are few [23, 35]. Differences in outcome between modalities or patient preference do not justify these variations [36]. Economic factors including financial and reimbursement strategy have been recognized as the main nonmedical factors in dialysis modality selection in countries around the world [34, 37, 38].

### 3.6. Implications of Health Economics on Peritoneal Dialysis Utilization

Economic evaluation is an analytical tool for decision making because it involves both cost and benefit which are being evaluated against each other [39]. A cost-effectiveness analysis includes life years gained, a cost utility analysis includes quality adjusted life years (QALY), and a cost benefit analysis includes monetary units as primary outcome, respectively [39]. Relevant perspectives to be considered include provider, payer, or society because in one situation items may be considered as costs but in other cases not [39]. For a proper evaluation, activity based costing (primary data) is the most appropriate cost approach to be carried out which could be resource intensive [40]. Costs are generally described in four categories: direct medical costs, direct nonmedical costs, indirect costs, and intangible costs [39]. Direct medical costs of dialysis include staff salaries, costs of dialyzers and extracorporeal circuits in HD, costs of solutions and disposables in PD, costs associated with radiology, laboratory, medications, capital costs of HD machines, and PD cyclers, costs of hospitalizations, and costs of outpatient consultations from other specialties. Direct nonmedical costs include building costs, facility utilities, and other overhead costs. Intangible costs are the costs associated with pain, suffering, and impairment in quality of life as well as the value of extending life. Intangible costs are often omitted from economic evaluations [39].

Some argue that economic evaluation such as cost-effectiveness or cost utility is only suitable to compare new against existing treatment. However, since economic factors influence dialysis provision, many countries conduct economic evaluation of HD and PD (both are established therapies). Economic evaluation studies are vital for health policy decisions. For example, in Thailand, the “PD-First” Policy has been promoted in 2008 as a model of initial treatment for ESRD patients under the Universal Coverage scheme [41] after the benefit of PD over HD has been shown in terms of medical expenses and cost-effectiveness [42]. Similarly, the government of Hong Kong developed the PD-First Policy based on its basis of cost-effectiveness [43]. Meanwhile, some other countries including Canada, China, Guatemala, India, Mexico, Spain, Taiwan, and the United States adopt a PD-Favored Policy. Cost-effectiveness plays a significant role on their policy decision [44]. Hong Kong has the highest proportion of PD in the world (72%) in 2013 [45]. HD remains an integral part of dialysis to cater for patients' preference, PD contraindicated patients, and transfer of PD to HD due to complications. In Malaysia, there is no clear PD-Favored Policy although PD is found to be marginally more cost-effective than HD. However, the acceptance and prevalence rate of PD treatment have increased twofold between 2004 and 2014 [12].

There are suggestions to revise the reimbursement structure in a way to provide incentives for home-based dialysis including PD and home HD to improve sustainability and patient outcome [34]. In the Canadian province of Ontario, the dialysis reimbursement system was changed from fee-for-service system with higher rates for in-centre HD to a modality-independent weekly capitation fee in 1998. PD use increased in Ontario while in the rest of Canada there were declines in PD use [46]. In Japan, reimbursement for dialysis was determined on a fee-for-service basis until 1999. Reimbursement for HD has been reduced based on recommendation by the Council on Economic and Fiscal Policy [47]. In Taiwan, there were four policies proposed by the Ministry of Health and Welfare relating to modality selection to increase the incidence of patients receiving PD as initial RRT [48]. In the US, the near 25-year-old fee-for-service payment method for dialysis was replaced by new prospective payment system (PPS). The new PPS places most costs for dialysis care, including injected medications within a bundle of services where the dialysis facility and the nephrologist both have financial incentives to promote PD [49]. There was an increased PD use in the 2-year period after the implementation of PPS [50].

The most frequently cited motivations for PD-First or PD-Favored Policies were to increase patient access to care, control costs through lower infrastructure and capital investments, empower patients, and optimize treatment provision [44]. This is particularly important in Malaysia as 97% of PD patients are being treated at public settings and there is geographical imbalance of dialysis access. PD is managed by salaried public-sector nurses and nephrologists and it is believed that more profits are obtained from private centre HD [51]. Recently, a budget impact analysis of HD versus PD was developed to estimate dialysis associated costs from Malaysian government perspective [52]. It was reported that increasing the use of PD for eligible patients could improve patients' access to dialysis in rural areas of Malaysia as the current funding model favors the setting up of HD centres in urban areas. Only government and NGOs are willing to open HD centres in less developed areas. Reliance on HD extensively in the current economic condition will further aggravate the healthcare budget [52].

Decision-analytical modeling in economic evaluation has been widely used to examine overall cost over time. Modeling may have low validity due to data simulations, but it remains an essential aspect of economic evaluation [53]. Several decision-analytical models are used including decision tree and Markov model, discrete event simulation, and mathematical modeling [52]. Studies conducted in UK [54], Austria [55], and Australia [56] using Markov modeling found that increasing the allocation of PD patients using simulated data versus current practice had resulted in significant cost savings from a payer perspective. Similar findings were reported in Malaysia through the budget impact analysis of PD versus HD [52].

## 4. Discussion

CKD is an important cause of death and disability but it remains asymptomatic till late stage when intervention cannot stop the progression of the disease [57]. Hence, detection and prevention of CKD at early stage are a necessity. Measures used to improve detection include training of nephrologists, urologists, and allied healthcare staff as well as continuous improvements in various allied services including radiology services, laboratory tests, and renal pathology [58]. CPG of management of CKD in adults was developed to assist in prevention and reduction in risk of CKD, screening and early detection of CKD, treatment of early CKD to prevent its progression to ESRD, and reduction in risk of cardiovascular disease [59]. Since diabetes and hypertension are two major risk factors to CKD in Malaysia, CPG of these diseases were also developed with strong emphasis on CKD detection via screening of proteinuria, microalbuminuria, and serum creatinine level to determine GFR. Numerous initiatives have been undertaken by the government to promote lifelong wellness and healthy lifestyle among the community. The Malaysia Health Promotion Board* (MySihat)* was established by MOH in 2006 where the main objective is to set and develop the health promotion agenda across different sectors and settings particularly with the active participation of the NGOs.

However, the increasing incidence and prevalence of ESRD seem inevitable. Kidney transplantation is significantly associated with improved survival and quality of life, as well as substantial cost savings, compared with dialysis [21]. Malaysia has one of the lowest organ donation rates in the world [60] making dialysis the only viable option for majority of ESRD patients. Some patients, however, are managed by palliative care when dialysis support is not feasible or being withdrawn and the operational policy was established in 2010 [61]. Palliative care is an important aspect of medical service offered by some major hospitals in this country to minimize suffering by early identification, assessment, and prompt intervention of physical, psychosocial, and spiritual problems related to ESRD.

Dialysis is a costly treatment with substantial impact on healthcare budget especially when HD, a costlier treatment than PD, dominates dialysis provision. Malaysia's dialysis financing system (public-private partnership) is in alignment with WHO recommendation of health system and financing for RRT [21]. This review suggests that some countries tend to widen their PD utilization amid positive economic implications and improved dialysis access. Nevertheless, increasing PD utilization require strong coordinated effort. Liu et al. in their overview of the impact of PD-First or PD-Favored Policies around the world indicated that barriers to policy implementation are generally associated with government policy, economics, provider or healthcare professional education, modality-related factors, and patient-related factors [44]. Education and training for healthcare professionals including nephrologists, doctors, nurses, and other dialysis staff as well as patients' successful PD catheter placement and technique survival are crucial for its expansion [34, 62–65]. Collaboration between various departments such as the Ministry of Health, National Kidney Foundation (NKF), and other NGOs is vital to ensure expansion of the PD program. For example, in Thailand, improved survival rates and technique were observed after three-year PD-First Policy implementation. There was support from the National Health Security Office, the Nephrology Society of Thailand, the Dialysis Nurse Association, the Kidney Foundation of Thailand, and other NGOs [41]. Resource availability and reimbursement or incentives are important for these initiatives [34].

Reliance on an economic evaluation research conducted many years ago could be a concern. The cost of treatments has changed tremendously. Cost of HD is driven mainly by fixed costs of facility space and staff while cost of PD is determined mainly by variable costs such as solutions and disposables. There have been large reductions in the price of erythropoietin (EPO) albeit PD patients have a lower requirement for ESAs [12, 26, 27]. Salaries of dialysis staff have increased accompanied by rising costs of land, building, and utilities. There are also variations in costs of dialysis machines, dialyzers, medications, and dialysis consumables. Since most HD is conducted at hospital or clinic while PD is performed at home, the costs of both treatments could change significantly. The recent budget impact analysis of HD versus PD reported several limitations including reliance on previously conducted economic evaluation of dialysis and costs of hospitalizations being not included [52]. Hence, a current comprehensive global cost evaluation of PD and HD (either from provider or societal perspective) is vital for any potential changes in policy and reimbursement strategies.

## 5. Conclusion

In conclusion, increasing ESRD prevalence in Malaysia is imposing a heavy financial burden on the healthcare budget especially when kidney transplants are limited. This paper addresses the dialysis provision in Malaysia, issues and implications of health economics on PD utilization. The literature show that PD is more cost-effective than HD but PD is underutilized in many countries including Malaysia. Some countries adopt “PD-First Policy” or “PD Favored Policy” from economic perspectives ensuring patients' quality of care, outcomes and widening PD utilization. Finding sustainable health polices and reimbursement strategies is essential to contain spending on ESRD treatment and improve patients' disparities in access to dialysis through economic evaluation studies.

## Abbreviations

CAPD: Continuous Ambulatory Peritoneal Dialysis
CKD: Chronic kidney disease
CPG: Clinical practice guidelines
DALYs: Disability adjusted life years
EPO: Erythropoietin
ESA: Erythropoiesis Stimulating Agent
ESRD: End-stage renal disease
GDB: Global Burden of Disease
GDP: Gross domestic product
HD: Hemodialysis
MCO: Managed Care Organization
MDTR: Malaysian Dialysis and Transplant Registry
MOF: Ministry of Finance
MOH: Ministry of Health
NGO: Nongovernmental organization
PD: Peritoneal dialysis
PMP: Per million population
PPS: Prospective payment system
QALY: Quality adjusted life years
RRT: Renal replacement therapies
SOCSO: Social Security Organization
YLL: Years of life lost
WHO: World Health Organization.
